# Loss of WT1 Drives Adaptive Plasticity in CCDC6-RET Selpercatinib-Resistant Papillary Thyroid Cancer

**DOI:** 10.3390/cimb48030274

**Published:** 2026-03-04

**Authors:** Giuseppe Siragusa, Laura Tomasello, Mattia Biondo, Fabiola Vaglica, Carla Giordano, Giorgio Arnaldi, Giuseppe Pizzolanti

**Affiliations:** 1 Department of Biological, Chemical and Pharmaceutical Sciences and Technologies (STEBICEF), University of Palermo, 90128 Palermo, PA, Italy; giuseppe.siragusa01@unipa.it (G.S.); mattia.biondo@unipa.it (M.B.); 2 Department of Health Promotion, Mother and Child Care, Internal Medicine and Medical Specialties (PROMISE), University of Palermo, 90127 Palermo, PA, Italy; fabiola.vaglica@unipa.it (F.V.); carla.giordano@unipa.it (C.G.); giorgio.arnaldi@unipa.it (G.A.); giuseppe.pizzolanti@unipa.it (G.P.); 3 Advanced Technologies Network Center (ATEN Center), University of Palermo, 90128 Palermo, PA, Italy

**Keywords:** cancer, resistance, thyroid, selpercatinib, CCDC6-RET, WT1

## Abstract

**Background**: Papillary Thyroid Cancer (PTC) harboring CCDC6-RET translocation is typically classified as a differentiated epithelial tumor. Although Selpercatinib, a RET-selective drug, was recently approved for use in advanced PTC, the emergence of drug resistance has already been observed. Tumor plasticity, including non-canonical Epithelial–Mesenchymal Transition (EMT) programs, is recognized as a key mechanism underlying drug resistance. The downregulation of the transcription factor Wilms’ Tumor 1 (WT1) in cancer is associated with increased motility, invasiveness, and metastatic potential. **Methods**: In this study, we developed a selpercatinib-resistant PTC-derived cell line, TPC-1-SelpR. Bioinformatic analyses were conducted to study the promoter of the *CCDC6-RET* gene and the transcriptomic landscape of PTC from RNAseq data. Subsequent real-time PCR, Western blot, and imaging techniques, such as confocal microscopy (CM) and fluorescence microscopy (FM), were employed to study the effects of WT1 loss-of-function following RNAi silencing. **Results**: In TPC-1-SelpR, WT1 expression appears downregulated compared to its counterpart, TPC-1. Crucially, WT1 silencing induced a context-dependent modulation of the CCDC6-RET driver: while WT1 silencing reduced CCDC6-RET expression in TPC-1, in TPC-1-SelpR, a post-transcriptional compensation of CCDC6-RET was observed. The gene expression of several factors involved in EMT, such as Twist, Vimentin, Integrin beta-1, and Profilin, was rewired in TPC-1-SelpR^WT1-knockdown^. Although the Vimentin protein product decreased, CM and FM analyses confirmed a reorganization of residual protein: the subcellular redistribution was more dispersed in TPC-1-SelpR^WT1-knockdown^. Further upregulation of the stemness factor Sox2 over the differentiation factor Sox17 occurred. These molecular changes were associated with higher cell motility of TPC-1-SelpR^WT1-knockdown^. **Conclusions**: Collectively, these findings suggest that WT1 is a critical regulator involved in tumor plasticity, thereby supporting selpercatinib resistance.

## 1. Introduction

Papillary thyroid cancer (PTC) is the most common endocrine disease, and its incidence is constantly increasing [1]. Most thyroid tumors originate from follicular epithelial cells and can be divided into papillary, follicular, poorly differentiated, and anaplastic types. Among these, PTC is a type of differentiated thyroid tumor (DTC) [2]. The management of DTCs mainly follows surgical excision, which is effective, and in case of recurrence risk, radioactive iodine therapy is employed; in advanced cases, when there is no longer a response to radioactive iodine, molecular tests are performed to identify key mutations, which act as drivers in this context [3]. Vandetanib and cabozantinib, approved by the Food and Drug Administration (FDA), are effective for the treatment of fusion-positive DTCs, including PTCs harboring CCDC6-RET. However, their effectiveness is limited by the lack of selectivity against REarranged during Transfection (RET). In this regard, sometime later, in 2020, the drugs selpercatinib and pralsetinib, highly selective inhibitors, were approved [4]. Selpercatinib, occupying both the front and back pockets, interacts with the active cleft of RET [5] by inhibiting the protein activity of the CCDC6-RET translocation product. However, the emergence of acquired resistance necessitates identifying new mechanisms of drug evasion [6]. Regarding functional mechanisms, CCDC6 plays important roles in cell biology. Coiled-Coil Domain Containing 6 (CCDC6) protein is stabilized after DNA damage in the Ataxia-Telangiectasia Mutated (ATM)-assisted priming system, resulting in the maintenance of the cell cycle checkpoint and DNA homology repair mechanisms [7]. In PTC harboring the CCDC6-RET fusion product, the recombination event resulting in the fusion of the sequences AGCGTGACCATC/GAGGATCCAAAG, belonging, respectively, to part of exon 1 of CCDC6 and 12 of RET, generates the translocation product CCDC6-RET, which, in fact, makes the *RET* gene constitutively expressed [8]. To date, cases of on-target resistance have been reported in other RET-fusion-positive tumors, and not in PTC. Nevertheless, off-target mechanisms involving RAS and BRAF in both NSCLC and PTC, as well as alterations in different signaling pathways that lead to tumor plasticity, exist [6,9]. Although it is a differentiated tumor, PTC acquires RET-dependent function, and its continued inhibition by selpercatinib leads to resistance, suggesting that the underlying mechanisms of tumor plasticity may be involved. EMT is one of the major mechanisms involved in the progression of epithelial tumors, including PTC [10], and several factors govern EMT, both physiologically and pathologically. Several proteins are involved in maintaining cellular architecture during EMT: Vimentin, a core cytoskeletal protein especially in mesenchymal cells, is generally upregulated during EMT [11,12]; Integrin beta-1 and Profilin support mechanotransduction and survival [13,14,15]. The expression of these proteins is regulated by several transcription factors, among them Twist, a crucial transcription factor expressed in Mesenchymal Stem Cells (MSCs) derived from mesoderm [16], which is involved in the induction of EMT [17]. Oct4 and Sox2 are two transcription factors that generally maintain stemness-like identity in cancer [18,19,20,21], whereas Sox17 appears to be involved in differentiation at the embryonic level. In cancer, Sox17 acts as a tumor suppressor, inhibiting EMT [22,23]. WT1, by regulating the expression of a multitude of genes, plays fundamental roles in developmental biology, particularly in maintaining the balance between epithelial and mesenchymal identities during EMT [24]. In the kidney, WT1 is involved in the development of Wilm’s tumor. During embryonic development, WT1 is required for metanephric mesenchyme formation, and in adult tissues, it is involved in tissue homeostasis [25,26], although its expression is relatively low [27]. In cancer, as in EMT, it can act as both an oncogene and a tumor suppressor, depending on the context. In multiple myeloma, WT1 mRNA expression levels may be normal and low in a subtype of Acute Myeloid Leukemia (AML) characterized by a distinct pattern of mutations [28]. In AML, WT1 is implicated as a key hub of epigenetic plasticity, primarily involved in drug resistance. WT1 interacts with beta-catenin, leading to convergent cellular phenotypes [29]. In breast cancer, WT1 expression alters the epithelial/mesenchymal balance: the knockdown of WT1 increases motility, invasiveness, and metastatic potential [30]. In PTC, its expression appears increased and promotes tumor progression [31], suggesting that both high and low levels of WT1 may be required in different contexts. Over the past few years, cancer research has included the study of several nutraceuticals with antitumor activity, contributing to a deeper understanding of the mechanisms by which they exert these functions. The nutraceuticals berberine and shikonin are closely related to RET and WT1 signaling, through different mechanisms. Berberine interacts with the RET promoter and, by stabilizing the RET G-quadruplex, downregulates *RET* gene expression; however, it is unable to modulate CCDC6-RET expression, as the latter is driven by the CCDC6 promoter, which differs from that of RET [32]. A molecular docking model shows the formation of a Shikonin-WT1 complex, and further experiments have established its ability to reduce WT1 protein expression in a dose-dependent manner, likely by decreasing its half-life [33]. Both berberine [34,35,36,37] and shikonin [38,39,40] are effective in the context of chemotherapy resistance, sensitizing resistant cells through several mechanisms.

## 2. Materials and Methods

### 2.1. Cell Cultures and Development of the TPC-1-SelpR Cell Line

The TPC-1 cell line harboring the CCDC6-RET translocation, purchased by Cytion (Nikola-Tesla-Str 3, Heidelberg, Germany), was grown in adherence in the presence of Roswell Park Memorial Institute medium (RPMI) high glucose medium (EuroClone Spa, Milan, Italy), 10% fetal bovine serum (FBS; EuroClone Spa, Milan, Italy), and 1% penicillin-streptomycin. The medium was replaced every 3 days until the cells reached confluence. To develop the TPC-1-SelpR cell line, the cells were treated with selpercatinib for approximately 4 months, starting at a dose equivalent to the IC_50_. This drug dose was then gradually increased up to 4 µM until the cells appeared less sensitive to selpercatinib.

### 2.2. Doubling Time

To calculate the doubling time of TPC-1 or TPC-1-SelpR, the same number of cells (60,000) were plated in a six-well plate. Cells were counted at 48 h and 120 h. Subsequent analyses were performed by Graphpad Prism 5, using the “Exponential Growth Equation” function.

### 2.3. Selpercatinib, Berberine, Shikonin, and Vandetanib

Selpercatinib (lot #240149) and Shikonin (lot #654399) were purchased from MedChemExpress (MCE^®^) (1 Deer Park Dr, Suite F, Monmouth Junction, NJ, USA) as powders and resuspended in DMSO. Berberine (lot # SLBG1303V) was purchased from Sigma-Aldrich Merck (Darmstadt, Germany) as a powder and resuspended in DMSO. Vandetanib was purchased from Selleckchem (Milan, Italy) as a powder and resuspended in DMSO.

### 2.4. MTT Assay and IC_50_ Determination

To determine IC_50_ values, the MTT (3-(4,5-dimethylthiazol-2-yl)-2,5-diphenyltetrazolium) assay was used. TPC-1 and TPC-1-SelpR were seeded at densities of 1.8 × 10^3^ and 3.6 × 10^3^, respectively, in 96-well plates. Briefly_,_ several dose–response curves were performed with increasing drug concentrations, starting at 1000 nM (for selpercatinib and vandetanib) and 100 µM (for berberine and shikonin), in successive serial dilutions. The cells were cultured up to 72 h. The absorbance was measured at 570 nm using a CLARIOstar spectrophotometer (BMG Labtech, Ortenberg, Germany). All analyses were conducted three days after the respective treatments. The dose–response curve was fitted using the Four Parameters Logistic (4PL) model in GraphPad Prism (GraphPad Software, La Jolla, CA, USA) to determine the IC_50_ values.

### 2.5. Bioinformatic Analyses

To validate WT1 expression in PTC subjects, an ex vivo cross-analysis was performed on publicly available datasets. Clinical and molecular data from the subjects were obtained from the BioPortal for Cancer Genomics (https://www.cbioportal.org), investigating the TCGA Thyroid Carcinoma cohort. CCDC6-RET-positive samples (*n* = 16) with a diagnosis of PTC were filtered. To analyze the correlation between the presence of the CCDC6-RET translocation and WT1 expression levels, further analyses were conducted on The Human Protein Atlas (HPA, https://www.proteinatlas.org), and the data were expressed as pTPM (protein-coding transcripts per million). To identify potential transcription factors interacting with the CCDC6-RET promoter sequence, the Length-Aware Site Alignment Guided by Nucleotide Association (LASAGNA) [41] software algorithm was employed. Its operating principle involves aligning the Transcription Factor Binding Sites (TFBS) sequences, accounting for the lengths of the sites being investigated. After alignment, Position-specific Scoring Matrices (PSSMs) are constructed, which consider the content of individual bases and base pairs. LASAGNA-search then scans promoter sequences, assigning a score and *p*-value. The promoter of the human *CCDC6* gene was investigated over 500 base pairs (from −450 to +50). PTC transcriptome data were obtained by consulting the Gene Expression Omnibus (GEO) database (NCBI) for experiments conducted by Dai, L. et al., 2023 [42], accession GSE237841, ID 200237841. Briefly, four PTCs (accession numbers: GSM7655239, GSM7655240, GSM7655241, GSM7655242) and four adjacent healthy tissues (accession numbers: GSM7655235, GSM7655236, GSM7655237, GSM7655238) were considered. Differential Gene Expression (DGE) was performed using GEO2R, setting a cut-off of 0.05 with the Benjamini–Hochberg *p*-value correction. Finally, the upregulated genes (Magnitude Threshold: avg_log_2_FC ≥ 0.5; Direction: upregulated only) were selected in Excel. The upregulated genes previously obtained from GEO2R and Excel elaboration were processed with g:Profiler [43] to verify enrichments in different Gene Ontology (GO) classes (g:GOSt, Functional Profiling: Reac and TF were considered). The gene IDs of interest were entered, and the organism Human was selected. Statistically significant enrichments were identified using the g:SCS (Set Counts and Sizes) algorithm with a *p*-value threshold of <0.05.

### 2.6. MTS Assay

Cell viability was determined quantitatively by the MTS [3-(4,5-dimethylthiazol-2-yl)-5-(3-carboxymethoxyphenyl)-2-(4-sulfophenyl)-2H-tetrazolium] colorimetric assay using the MTS solution kit (REF G109A, Lot # 000506514, Promega, Madison, WI, USA) according to the protocol. TPC-1 and TPC-1-SelpR were seeded at a density of 1.8 × 10^3^ and 3.6 × 10^3^, respectively, in 96-well plates and treated with INTERFERin or INTERFERin-delivered siRNA or INTERFERin-delivered scrambled siRNA. The absorbance was measured at 490 nm using a CLARIOstar spectrophotometer, three days after the relative treatment.

### 2.7. Apoptosis Assay

To evaluate the potential apoptotic effect of WT1 silencing, an apoptosis assay was performed using the Cell Meter™ Phosphatidylserine Apoptosis Assay Kit (AAT Bioquest, Pleasanton, CA, USA). Briefly, TPC-1 and TPC-1-SelpR cells were seeded in 96-well black-wall/clear-bottom, and the test was performed according to the protocol. The green fluorescence intensity, reflecting phosphatidylserine externalization, was measured using a CLARIOstar spectrophotometer according to the protocol. Data were expressed as fold change relative to the controls.

### 2.8. RNA Extraction, Reverse Transcription, and Real-Time PCR

Total RNA was extracted from TPC-1 or TPC-1-SelpR cells using the RNeasy^®^ Mini kit (QIAGEN, Hamburg, Germany) according to the protocol. RNA absorbance was measured using a Nanodrop 2000 Spectrophotometer (Thermo Fisher Scientific, Waltham, MA, USA); concentrations were expressed in ng/µL. The purity of the RNA satisfied the A260/A280 ratio—between 1.9 and 2.1—and the A260/A230 ratio—between 2.0 and 2.2. A total of 1000 nanograms of RNA was reverse-transcribed as follows. Complementary DNA (cDNA) synthesis involved the following thermocycler program: 70 °C for 5 min, followed by a transition to 4 °C. The second step involves reverse transcriptase, buffer, MgCl_2_, and dNTPs, followed by incubation at 25 °C for 5 min and 42 °C for 60 min for elongation. Real-time PCR experiments used 10 ng of cDNA per reaction tube. The QuantiNova™ SYBR^®^ Green PCR kit included DNA polymerase, buffer, dNTPs, MgCl_2_, fluorescent Sybr green, and preselected primers. The two-step reaction involved initial denaturation at 95 °C for 2 min, followed by 35–40 cycles of denaturation at 95 °C for 5 s and annealing and extension at 60 °C for 10 s. To normalize the real-time PCR data, Beta-actin was chosen as the reference gene, and the ΔΔCt method was performed [44]. The primer sequences used for real-time PCR are listed below. For Beta-actin, forward is 5′-CCACACTGTGCCCATCTACG-3′, reverse is 5′-AGGATCTTCATGAGGTAGTCAGTCAG-3′. For WT1, forward is 5′-TGACTCTCCACTCCTCCTCA-3′, reverse is 5′-CAGGGCCTGTGAGTCAACTA-3′. For CCDC6-RET, forward is 5′-GCTGGAGACCTACAAACTGA-3′, reverse is 5′-CCTGACCACTTTTCCAAATTC-3′. Primers QuantiTect (QIAGEN): for Vimentin GeneGlobe ID: QT00095795; for Twist GeneGlobe ID: QT00011956; for Integrin beta-1 GeneGlobe ID: QT00068124; for Profilin GeneGlobe ID: QT00079520; for Oct4 GeneGlobe ID: QT00210840; for Sox2 GeneGlobe ID: QT00237601; for Sox17 GeneGlobe ID: QT00204099.

### 2.9. WT1 Knockdown Using siRNA

To silence the gene expression of the WT1 transcription factor, the RNA interference (RNAi) mechanism mediated by siRNA with the following sequences was used: sense sequence, 5′-GGACUGUGAACGAAGGUUU-3′; antisense sequence, 5′-AAACUUCGUUCACAGUCC-3′. The sense sequence was obtained from the study by Wang, X. et al. (2013) [45]. Regarding the experiments, the final siRNA concentration was 1 nM. The scrambled control, sc-36869 (Santa Cruz, CA, USA), was at 1 nM. The transfection of the relative siRNA and Scrambled was carried out using INTERFERin^®^ (Polyplus, 75 Rue Marguerite Perey, Illkirch, France), according to the protocol.

### 2.10. Immunofluorescence Microscopy Analyses

Fluorescence microscopy experiments were conducted in chambers or in 96-well plates to detect and evaluate fluorescence from Vimentin expression. Three days after gene silencing, the culture medium was removed, and the cells were washed twice in PBS to remove residual medium for immunofluorescence. This step was followed by fixation in paraformaldehyde (2%) for 20 min at room temperature. The cells were then permeabilized with Triton X-100 (0.1%) for 2 min, followed by blocking of nonspecific antigens with FBS (5%) for 45 min. Incubation with the primary antibody Vimentin (sc-7557, lot # K3012, Santa Cruz Biotechnology, Dallas, TX, USA) took place at 4 °C overnight, followed by incubation with the conjugated secondary antibody (Alexa Fluor^®^ 488 rabbit anti-goat IgG, ref A11078, lot # 1757125, Life Technology, Foster City, CA, USA). The cells were observed under a ZEISS LSM 910 (ZEISS, Oberkochen, Germany), equipped with Airyscan2 super-resolution, Dynamic Profiler, and Lightfield4D instant volumetric imaging module, and a Nikon Eclipse Ts2R microscope (Nikon, Tokyo, Japan) using FITC and DAPI filters.

### 2.11. Analysis with Fiji, an ImageJ Processing Package

To quantify Vimentin protein distribution in both TPC-1 and TPC-1-SelpR, the Fiji package (1.54p version) was used. The distribution was quantified by considering the dispersion area of Vimentin expression. The dispersion index was calculated using the ‘Analyze Particles’ tool as the ratio between Integrated Density (IntDen) and area, with results expressed as fold changes relative to the TPC-1 control group. 4 × 10^3^ cells for the condition were analyzed.

### 2.12. Wound Healing Assay

Cell motility was assessed by the scratch assay. TPC-1 or TPC-1-SelpR cells were grown in 24-well plates and treated with scrambled siRNA or WT1 siRNA three days before performing the migration assay to observe the effects of gene loss of function. Cells were observed and imaged using a Leica DMI 300B microscope (Leica, Wetzlar, Germany). Migration capacity was calculated as the ratio of the TPC-1 or TPC-1-SelpR cell closure area induced by WT1 siRNA, normalized by the closure area induced by the respective scrambled siRNA, at 24 h. The ratio was normalized as a fold change compared to the scrambled siRNA. The analysis was performed using ImageJ (1.54p version).

### 2.13. Protein Extraction

Cells were collected following the scraping in phosphate-buffered saline (PBS, Euroclone S.p.A, Pero, Italy), and centrifuged at 1200 rpm for 10 min at 4 °C. Subsequently, the cell pellets were resuspended in RIPA Lysis Buffer System (sc-24948, Santa Cruz Biotechnology, Inc., Dallas, TX, USA). The incubation on ice for 30 min followed. Lysates were then centrifuged at 14,000× *g* for 10 min at 4 °C. Protein concentration was determined using the Quick Start^TM^ Bradford 1× Dye Reagent (Bio-Rad, Hercules, CA, USA), and absorbance was measured with a BioPhotometer (Eppendorf, Hamburg, Germany).

### 2.14. Western Blot

20 µg of total protein from TPC-1 lysates were prepared under reducing conditions by adding 10× Sample Reducing Agent and 4× Bolt^TM^ LDS Sample Buffer (Invitrogen, Thermo Fisher Scientific, Waltham, MA, USA), followed by denaturation at 95 °C for 5 min. Samples were then loaded onto 4–20% Mini-PROTEAN^®^ TGX^TM^ gels (Bio-Rad, Hercules, CA, USA) and separated by electrophoresis at 150 V in Tris/Glycine/SDS running buffer (1×, Bio-Rad, Hercules, CA, USA). The protein transfer onto membranes was carried out using Trans-Blot^®^ Turbo Transfer Packs (Bio-Rad, Hercules, CA, USA) for 3 min at 2.5 A and 25 V with the Trans-Blot^®^ Turbo Transfer System (Bio-Rad, Hercules, CA, USA). Transfer efficiency was verified by brief staining with Ponceau S solution (0.2% Ponceau S in 3% trichloroacetic acid), followed by destaining in distilled water. After blocking in Tris-buffered saline containing 0.05% Tween-20 (T-TBS) supplemented with 1× Pierce^TM^ Clear Milk Blocking Buffer (Thermo Fisher Scientific, Waltham, MA, USA) for 1 h at room temperature. The membranes were incubated overnight at 4 °C with the following primary antibodies: anti-RET (EPR2871, ab134100, Abcam, Cambridge, UK), anti-Vimentin (sc-7557, Santa Cruz Biotechnology), anti-Sox2 (sc-365823, Santa Cruz Biotechnology), anti-Sox17 (sc-130295, Santa Cruz Biotechnology), anti-Oct4 (sc-5279, Santa Cruz Biotechnology), and anti-Beta-actin (sc-130301, Santa Cruz Biotechnology). The membranes were washed in T-TBS and incubated for 1 h at room temperature with the following secondary antibodies: mouse anti-rabbit IgG-HRP (sc-2357, Santa Cruz Biotechnology), mouse anti-goat IgG-HRP (sc-2354, Santa Cruz Biotechnology), m-IgGk BP-HRP (sc-516102, Santa Cruz Biotechnology). After TBS washing, antigen–antibody complexes were detected by using the SuperSignal^TM^ West Pico Plus Chemiluminescent Substrate (Thermo Fisher Scientific, Waltham, MA, USA). Chemiluminescent signals were acquired with the ChemiDoc^TM^ XRS imaging system (Bio-Rad, Hercules, CA, USA) and analyzed using Quantity One (4.6.3 version) software (Bio-Rad Laboratories, Hercules, CA, USA).

### 2.15. Statistical Analyses

All experiments, except bioinformatics, were performed in triplicate, and the data were expressed as means and SD. Analysis of variance (ANOVA) was conducted using GraphPad Prism 5, and a *p*-value ≤ 0.05, with a 95% confidence interval, was considered significant (* *p* < 0.05, ** *p* < 0.01, *** *p* < 0.001).

### 2.16. Abbreviations

TPC-1 knockdown for WT1 = TPC-1^WT1-kd^; TPC-1-SelpR knockdown for WT1 = TPC-1-SelpR^WT1-kd^.

## 3. Results

### 3.1. Development of the TPC-1-SelpR Cell Line as a Model of Selpercatinib-Resistant TPC-1

**Figure 1:**
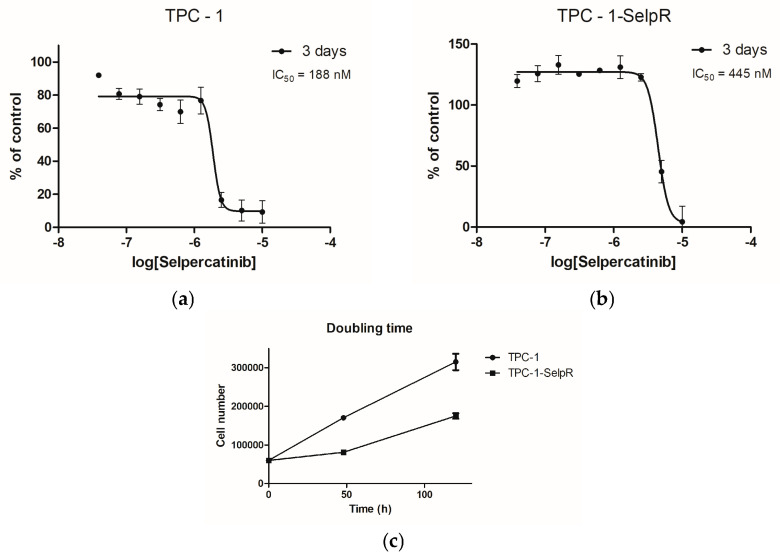
Effect of selpercatinib on cell viability and determination of the half-maximal inhibitory concentration (IC_50_) in TPC-1 (**a**) and TPC-1-SelpR (**b**) cell lines. Doubling Times of TPC-1 and TPC-1-SelpR cell lines (**c**).

The TPC-1 cell line was treated for four months with increasing doses of selpercatinib to induce drug resistance and create an in vitro model for further analyses. The drug proved highly effective even at low doses, compromising cell viability. Subsequent MTT analyses showed an IC_50_ of ~188 nM for the TPC-1 cell line (Figure 1a) and a significant increase in IC_50_ to 445 nM for the TPC-1-SelpR cell line (Figure 1b) three days after treatment, establishing the acquisition of resistance to selpercatinib. The doubling time was 63.22 h in TPC-1 and 71.75 h in TPC-1-SelpR (Figure 1c), suggesting that the proliferative index decreases in selpercatinib-resistant cells.

### 3.2. Ex Vivo WT1 Gene Expression Inversely Correlates with CCDC6-RET Translocation

**Figure 2:**
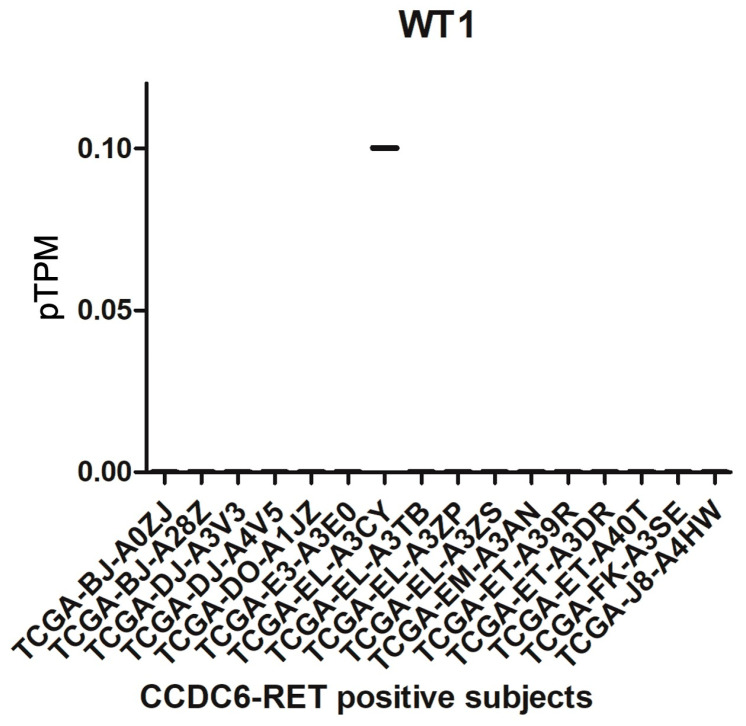
Analysis of WT1 gene expression in ex vivo PTC harboring CCDC6-RET translocation.

Analysis of a clinical cohort of *n* = 16 PTC patients harboring a CCDC6-RET translocation, obtained from the TCGA dataset, revealed that WT1 gene expression levels in pTPM were close to zero, suggesting an inverse correlation with the CCDC6-RET translocation (Figure 2).

### 3.3. The CCDC6-RET Promoter Contains WT1 Transcription Factor Binding Sites (TFBSs): Putative Role in CCDC6-RET Gene Expression Modulation

**Figure 3:**
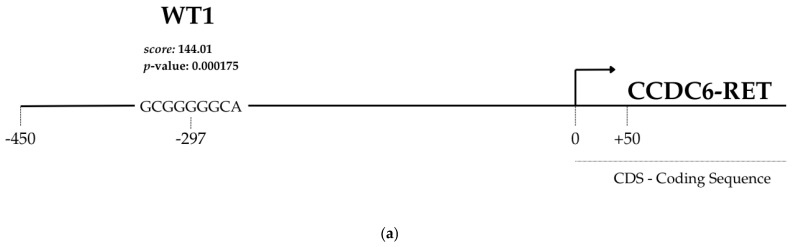

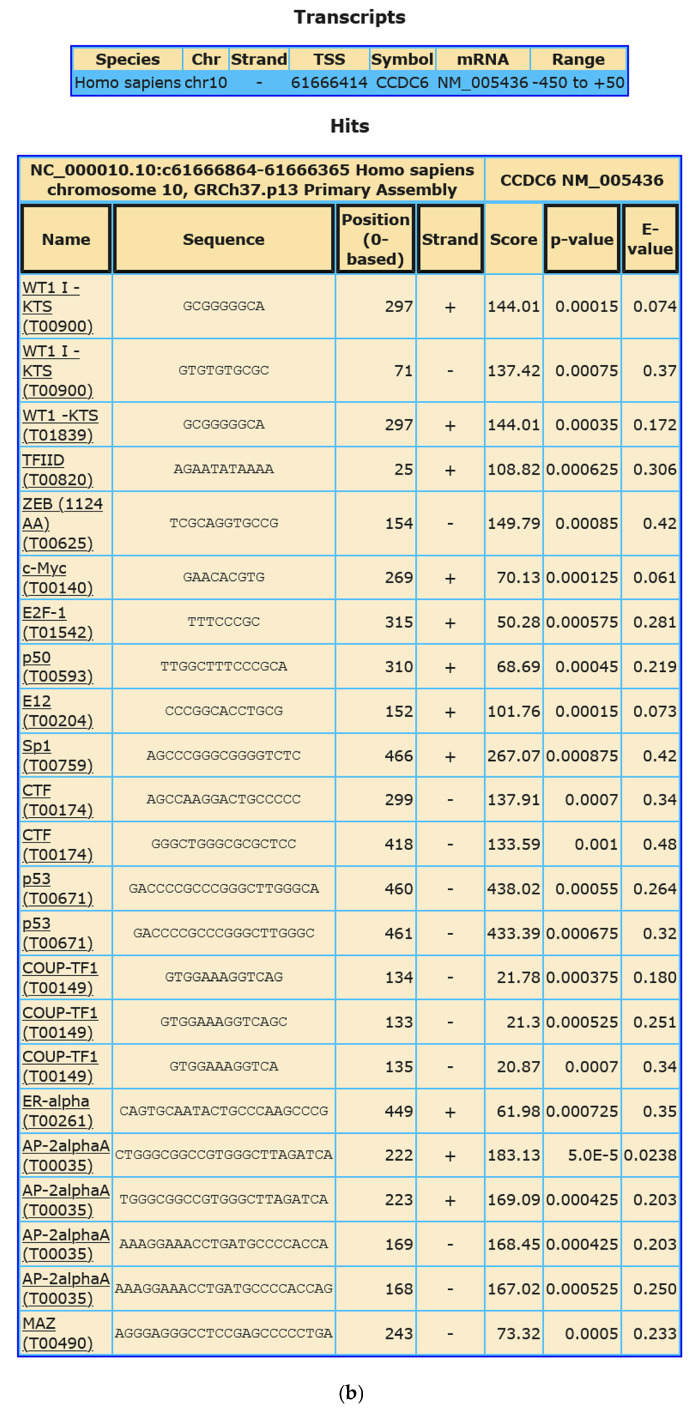

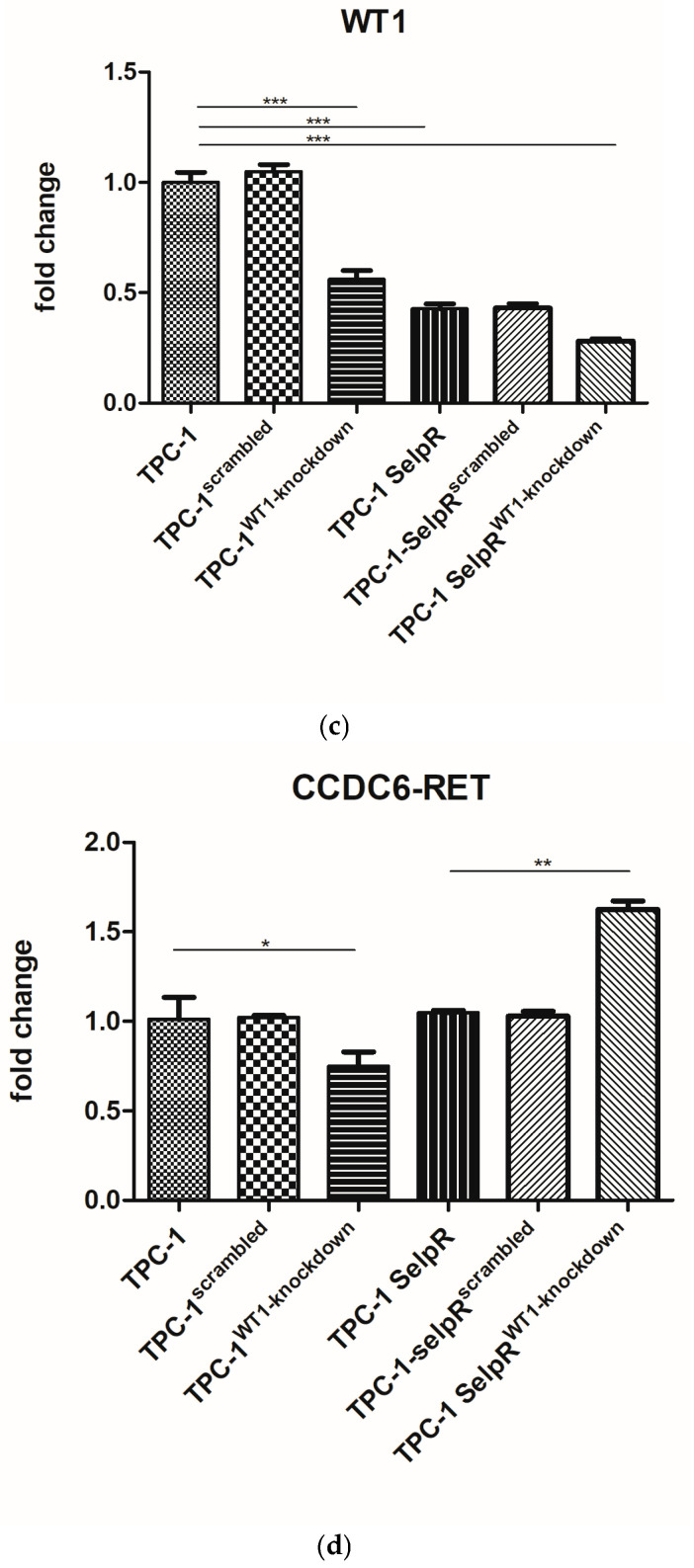
Graphical (**a**) and tabular (**b**) representation of the TFBSs on CCDC6-RET promoter of several transcription factors, including WT1. Real-time PCR results show downregulation of WT1 following siRNA treatment (**c**). Real-time PCR shows differential expression of CCDC6-RET following WT1 siRNA treatment in TPC-1 and TPC-1-SelpR (**d**) (* *p* < 0.05, ** *p* < 0.01, *** *p* < 0.001).

To evaluate whether a correlation exists between WT1 and CCDC6-RET, an in silico analysis using LASAGNA was performed. Specifically, several sequences are recognized by the WT1 transcription factor, as follows. The sequence GCGGGGGCA, located at 297 base pairs on the positive strand, has a 144.01 score and significant *p*- and *e*-values (0.00015 and 0.074). The sequence GTGTGTGCGC is present on the negative strand, located at 71 base pairs, has a 137.42 score, and significant *p*- and *e*-values (0.00075 and 0.037). The results of the in silico analysis suggest a strong functional correlation between the WT1 transcription factor and the CCDC6-RET promoter (Figure 3a,b). To confirm the in silico analysis, mRNA expression levels of WT1 and CCDC6-RET were assessed by real-time PCR in both TPC-1 and TPC-1-SelpR. The results of gene expression analyses suggest that WT1 is reduced by approximately 50% in the TPC-1-SelpR line compared to the control, TPC-1 (Fold decrease 0.42). Although resistant cells show reduced WT1 expression levels, they are not absent, as we found in TCGA. To evaluate the role of residual WT1, we analyzed the effects of further loss of function. We used a blunt siRNA, which, compared to a protruding-end siRNA, produces less effective silencing. siRNA directed against WT1 mRNA partially reduced its gene expression further, by approximately 50%, in TPC-1 and 75% in TPC-1-SelpR (Fold decrease 0.56 and 0.28, respectively) (Figure 3c) compared to TPC-1. Moreover, CCDC6-RET expression levels are similar in TPC-1 and TPC-1-SelpR (Fold change 1.15; 1.05). In TPC-1^WT1-kd^, CCDC6-RET gene expression is reduced compared to TPC-1 (Fold change 0.75 and 1.15, respectively). Conversely, in TPC-1-SelpR^WT1-kd^, its expression increases compared to the corresponding control (Fold change 1.62 and 1.05, respectively) (Figure 3d).

### 3.4. WT1 Knockdown Does Not Compromise Cell Viability of Resistant Cells

**Figure 4:**
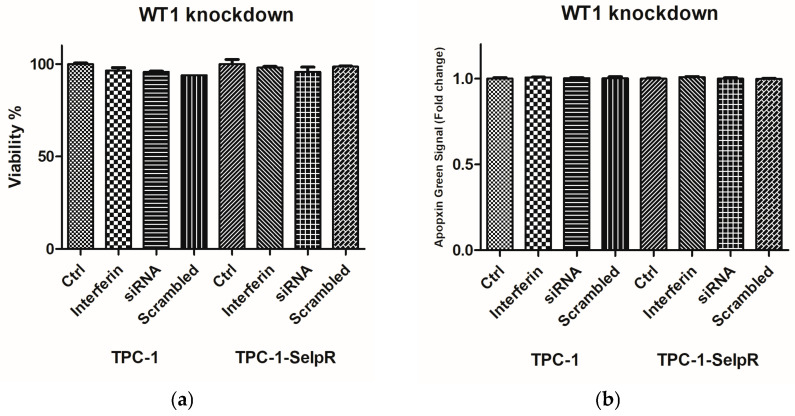
Treatment with siRNA against WT1 mRNA or scrambled control in TPC-1 and TPC-1-SelpR did not compromise cell viability (**a**) or stimulate apoptotic cell death (**b**).

To verify whether downregulation of WT1 affects cell viability, MTS and cell apoptosis assays were performed in TPC-1 and TPC-1-SelpR. MTS analyses confirmed that in TPC-1-SelpR^WT1-kd^, silencing did not compromise cell viability compared with the controls (Figure 4a). Cell apoptosis was assessed using the Cell Meter™ Phosphatidylserine Apoptosis Assay Kit. No significant increase in green fluorescence was revealed in both TPC-1 and TPC-1-SelpR, compared to controls (Figure 4b).

### 3.5. In Ex Vivo PTC, the Results of Gene Ontology (GO) Analyses Suggest Enrichments of Cell Motility- and WT1 Transcription Factor-Related Classes

**Figure 5:**
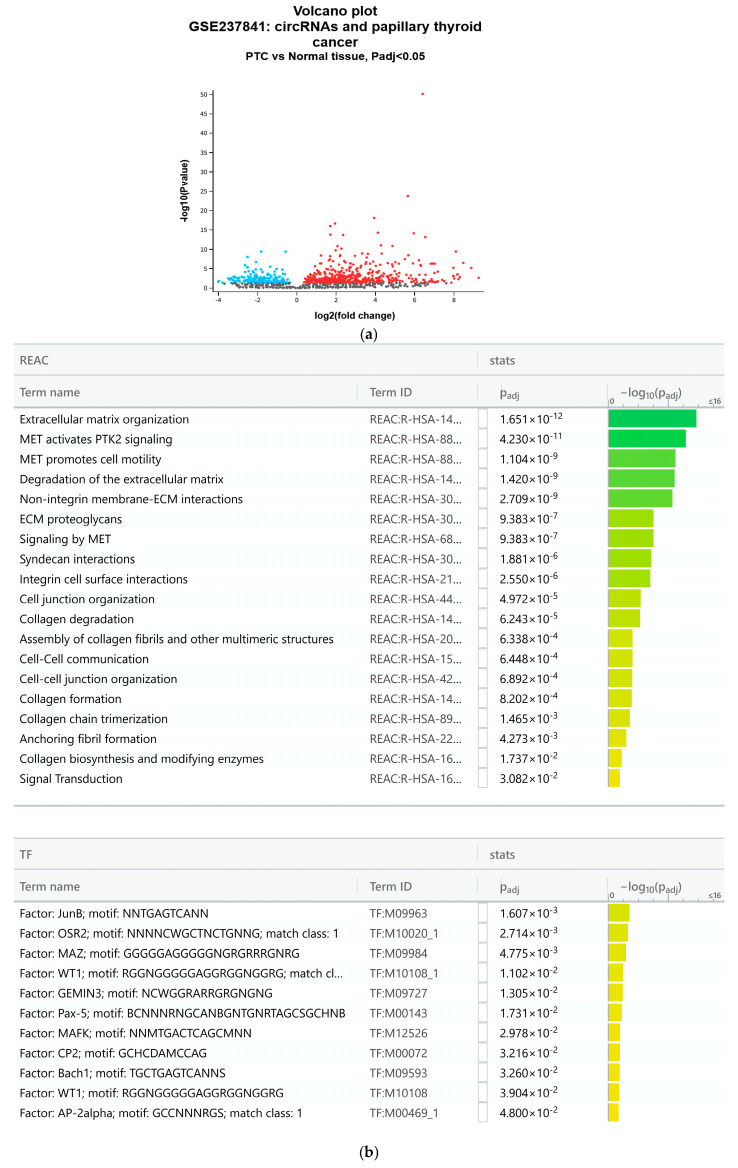
The upregulated genes were identified using GEO2R analyses following comparison of four PTC samples with adjacent healthy tissue; the results were represented as a volcano plot (**a**). The upregulated genes were filtered and processed with g:Profiler GO (**b**).

The GO analyses were conducted by identifying Differentially Expressed Genes (DEGs), resulting from a comparison of the PTC with adjacent healthy tissue. Expression profiling by high-throughput RNA sequencing identified all upregulated genes (Figure 5a). The g:Profiler algorithm showed enrichments for Integrin and signaling receptor binding classes, as well as for WT1, suggesting a strong functional correlation between WT1 and anchorage factors (the ID terms not shown in the figure belong to the REAC class) (Figure 5b).

### 3.6. The Further Downregulation of WT1 Suggests a More Plastic TPC-1 Phenotype

**Figure 6:**
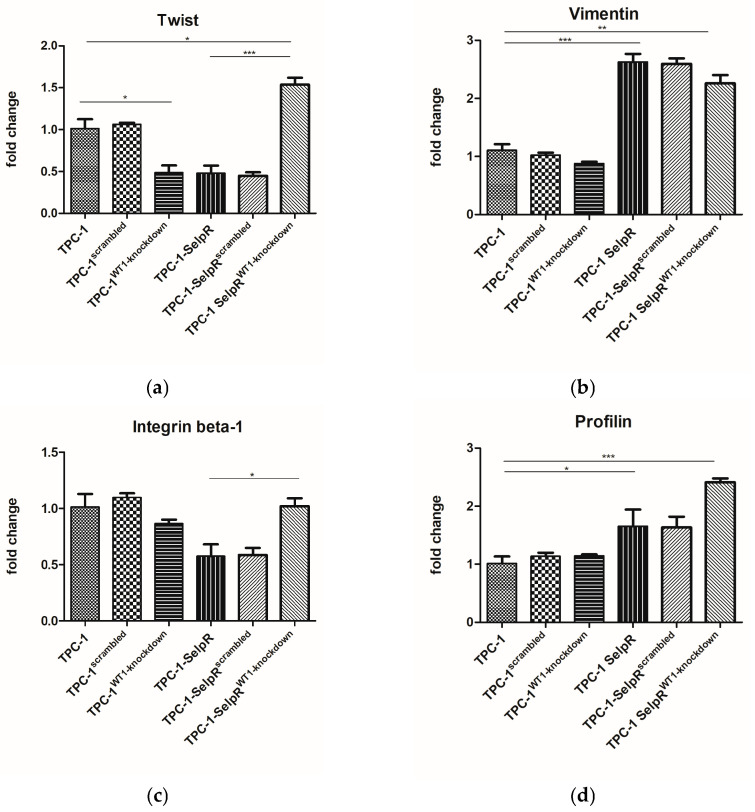
Real-time PCR results exhibit an increase in Twist (**a**), Vimentin (**b**), Integrin beta-1 (**c**), and Profilin (**d**) in TPC-1-SelpR when WT1 is further downregulated by siRNA treatment (* *p* < 0.05, ** *p* < 0.01, *** *p* < 0.001).

To investigate whether WT1 correlates with anchorage factors identified by GO analysis, several genes encoding the main proteins involved were assessed, including Twist, Vimentin, Integrin beta-1, and Profilin. Twist gene expression appears increased in TPC-1-SelpR^WT1-kd^ (Fold increase 1.53 vs. TPC-1) (Figure 6a). Vimentin gene expression is increased in TPC-1-SelpR (Fold increase 2.74 vs. TPC-1) and remains increased in TPC-1-SelpR^WT1-kd^ (Fold increase 2.26 vs. TPC-1) (Figure 6b). Integrin beta-1 increases in TPC-1-SelpR^WT1-kd^, in comparison with TPC-1-SelpR (Figure 6c). Profilin is increased in TPC-1-SelpR (Fold increase 1.65 vs. TPC-1) and in TPC-1-SelpR^WT1-kd^ (Fold increase 2.41 vs. TPC-1) (Figure 6d).

**Figure 7:**
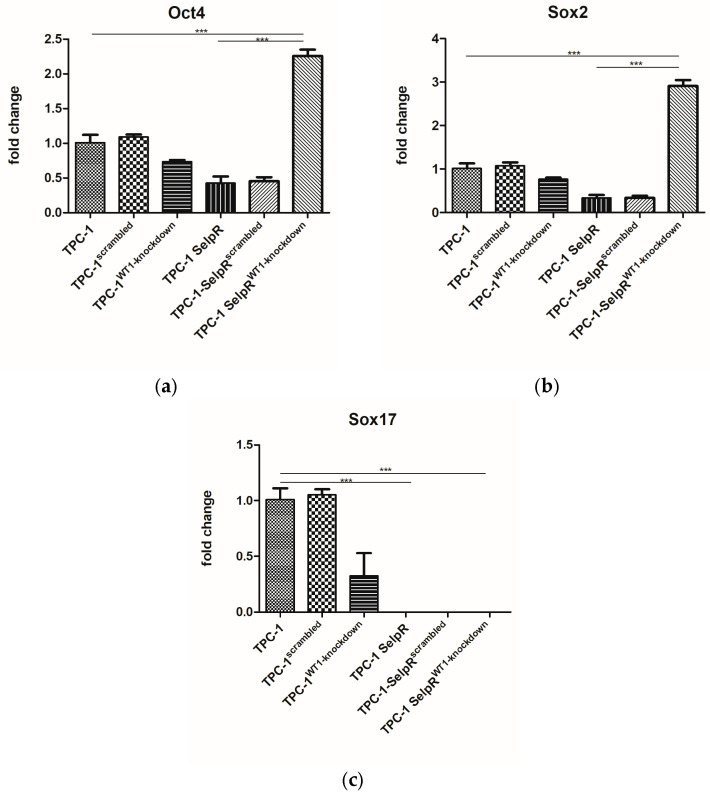
Real-time PCR results exhibit an increase in Oct4 (**a**) and Sox2 (**b**) expression, when Sox17 (**c**) is undetectable (*** *p* < 0.001).

Since higher motility correlates with cancer stem-like features, the expression of the main stem transcription factor was assessed. Oct4 and Sox2 generally maintain stemness-like identity in cancer, whereas Sox17 promotes a more epithelial, differentiated phenotype. Gene expression analysis revealed that in TPC-1-SelpR^WT1-kd^, Oct4 and Sox2 gene expression was found to increase (Fold increase 2.25 and 2.90 vs. TPC1) (Figure 7a,b), whereas Sox17 was undetectable compared to TPC-1 (Figure 7c).

### 3.7. Confocal Microscopy Analyses Reveal Vimentin Structure Size and Subcellular Redistribution in TPC-1-SelpR

**Figure 8:**
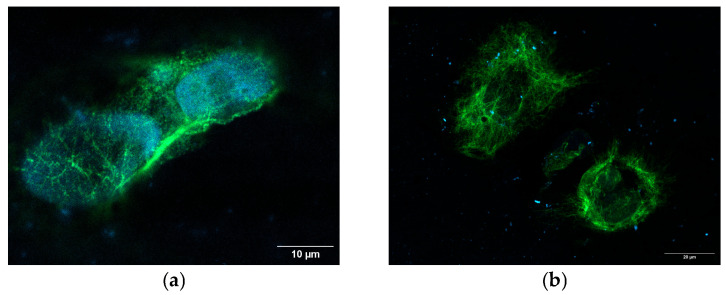
Confocal microscopy analyses revealing Vimentin expression in TPC-1 (**a**) and TPC-1-SelpR (**b**) (Vimentin ~ green fluorescence, DAPI ~ blue fluorescence).

To verify the distribution of the Vimentin protein product in TPC-1-SelpR, confocal microscopy analyses were conducted. Subsequent measurement of the area covered by the Vimentin signal revealed that, when compared to TPC-1 (Figure 8a), it is more diffused in TPC-1-SelpR (Fold increase 3.2) (Figure 8b).

### 3.8. Immunofluorescence Analyses of Cell Population Confirmed Morphological Changes in Resistant Cells Following WT1 Knockdown

**Figure 9:**
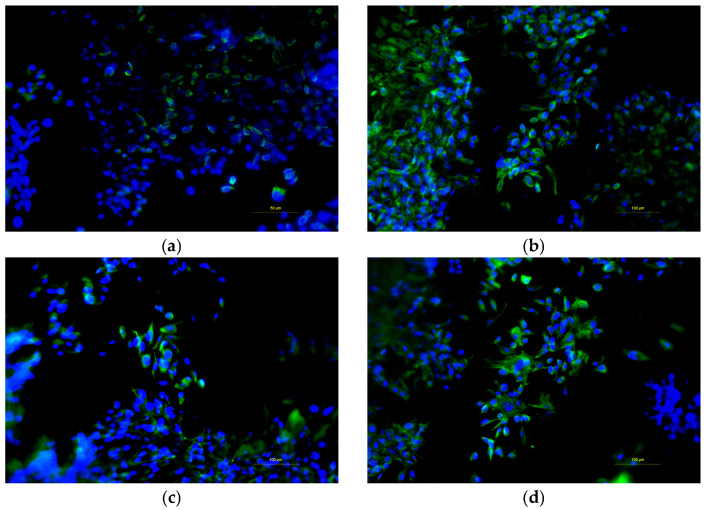
Fluorescence microscopy analyses show Vimentin protein product in TPC-1 (**a**), TPC-1^WT1-kd^ (**b**), TPC-1-SelpR (**c**), and TPC-1-SelpR^WT1-kd^ (**d**) (Vimentin ~ green fluorescence, DAPI ~ blue fluorescence).

A series of immunofluorescence analyses was conducted to evaluate morphological changes using Vimentin. As shown, Vimentin expression is visible in green in TPC-1 (Figure 9a), TPC-1^WT1-kd^ (Figure 9b), TPC-1-SelpR (Figure 9c), and TPC-1-SelpR^WT1-kd^ (Figure 9d). Single-cell analyses conducted using Fiji allowed us to calculate a series of morphological data. The area covered by the Vimentin signal is greater in TPC-1^WT1-kd^ (Fold increase 3.63), TPC-1-SelpR (Fold increase 3.58), and TPC-1-SelpR^WT1-kd^ (Fold increase 2.59) when compared to TPC-1. The number of Vimentin-positive particles is increased in TPC-1^WT1-kd^ (Fold increase 6.21), TPC-1-SelpR (Fold increase 3.28), and TPC-1-SelpR^WT1-kd^ (Fold increase 4.08). The density of Vimentin expression per single cell decreases in TPC-1^WT1-kd^ (Fold decrease 0.58) and TPC-1-SelpR^WT1-kd^ (Fold decrease 0.63), suggesting that Vimentin appears more dispersed when WT1 is downregulated.

### 3.9. siRNA-Mediated Knockdown Context-Dependently Regulates Wound Healing in TPC-1 and TPC-1-SelpR

**Figure 10:**
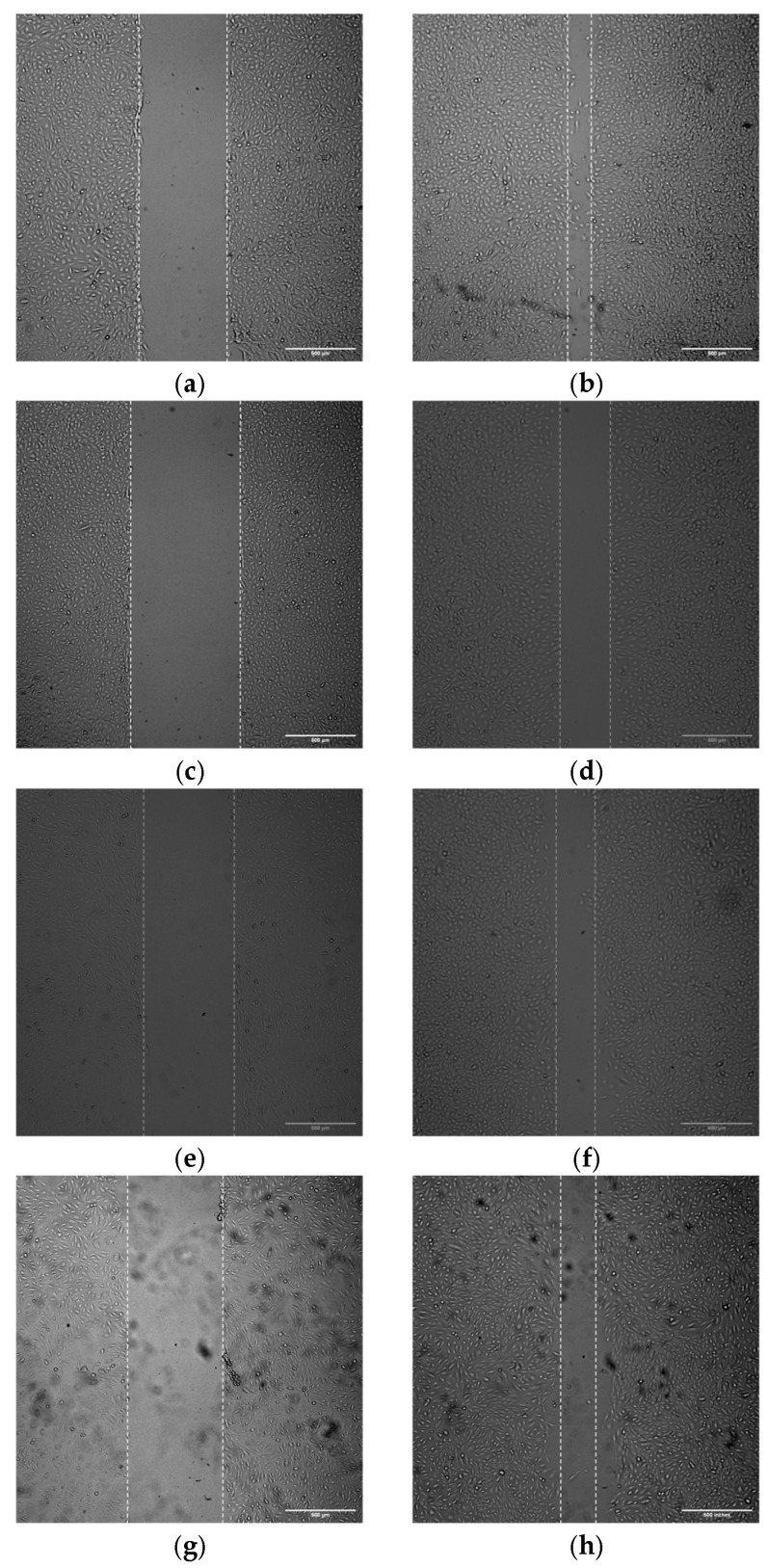
Wound healing assay in (**a**) TPC-1^scrambled^ at 0 h; (**b**) TPC-1^scrambled^ at 24 h; (**c**) TPC-1^WT1-kd^ at 0 h; (**d**) TPC-1^WT1-kd^ at 24 h; (**e**) TPC-1-SelpR^scrambled^ at 0 h; (**f**) TPC-1-SelpR^scrambled^ at 24 h; (**g**) TPC-1-SelpR^WT1-kd^ at 0 h; (**h**) TPC-1-SelpR^WT1-kd^ at 24 h.

**Figure 11:**
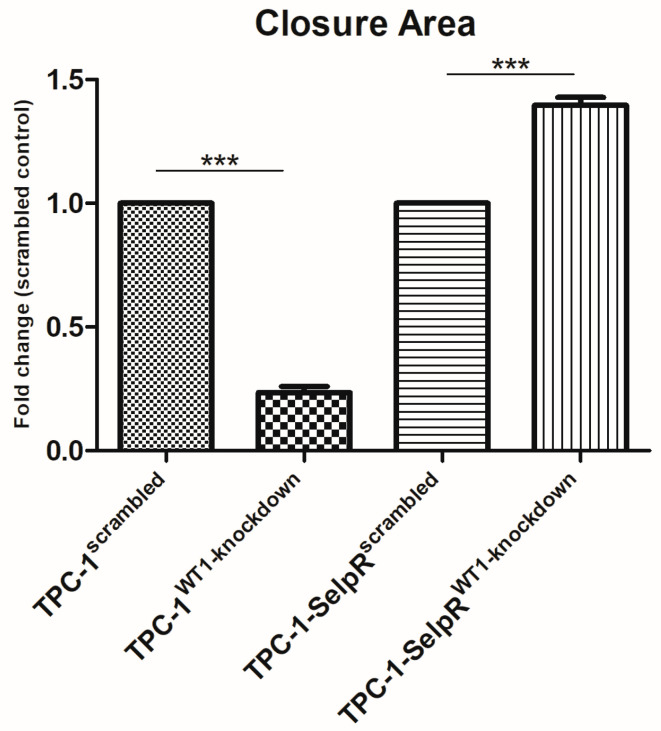
Wound healing assay at 24 h, compared to the respective controls as fold changes (*** *p* < 0.001).

To study the effect of WT1 downregulation on the migration of selpercatinib-resistant cells, both cell lines were seeded in 24-well plates, and wound-healing assays were performed at 24 h. The ability to cover the cell-depleted area was reduced in TPC-1^WT1-kd^ compared to its scrambled siRNA-treated control (Fold decrease 0.23) (Figure 10a–d). Conversely, in TPC-1-SelpR, transfection with siRNA against WT1 induced a greater ability to cover the wound, compared to TPC-1-SelpR treated with the scrambled control (Fold increase 1.39) (Figure 10e–h). Specifically, the ratio is shown in Figure 11.

### 3.10. Protein Profile Reveals a Stem-like Commitment

**Figure 12:**
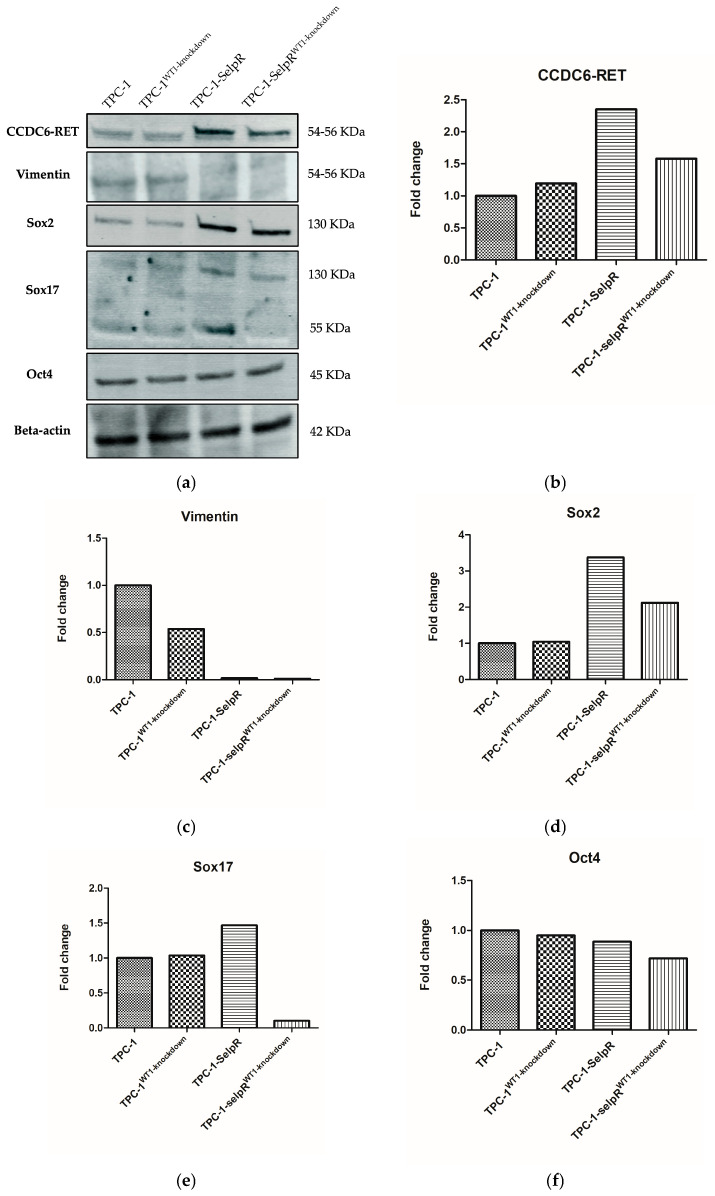
Western blot (**a**) and densitometric analyses of CCDC6-RET (**b**), Vimentin (**c**), Sox2 (**d**), Sox17 (**e**), Oct4 (**f**), expressed as fold change of TPC-1.

Further analyses moved from transcriptional to post-transcriptional context. Specifically, we first assessed CCDC6-RET expression. Western blot analyses (Figure 12a) revealed that the fusion product is upregulated in TPC-1-SelpR (Fold increase 2.35) and in TPC-1-SelpR^WT1-kd^ (Fold increase 1.58) when compared to TPC-1. When compared to TPC-1-SelpR, the fusion product protein expression decreases in TPC-1-SelpR^WT1-kd^ (Fold decrease 0.67) (Figure 12b). In TPC-1-SelpR^WT1-kd^, although the Vimentin protein product is almost absent (when compared to TPC-1) (Figure 12c), the Sox2 product (Figure 12d) predominates over that of Sox17 (Figure 12e) (Sox2/Sox17 ratio = 6.96). Oct4 expression remains almost unchanged in both TPC-1 and TPC-1-SelpR and slightly decreases in TPC-1-SelpR^WT1-kd^ (Fold decrease 0.8) (Figure 12f).

### 3.11. Exploratory Analyses, Cross-Resistance, and Collateral Sensitivity: Differential Sensitivity of TPC-1 and TPC-1-SelpR to Berberine, Shikonin, and Vandetanib

**Figure 13:**
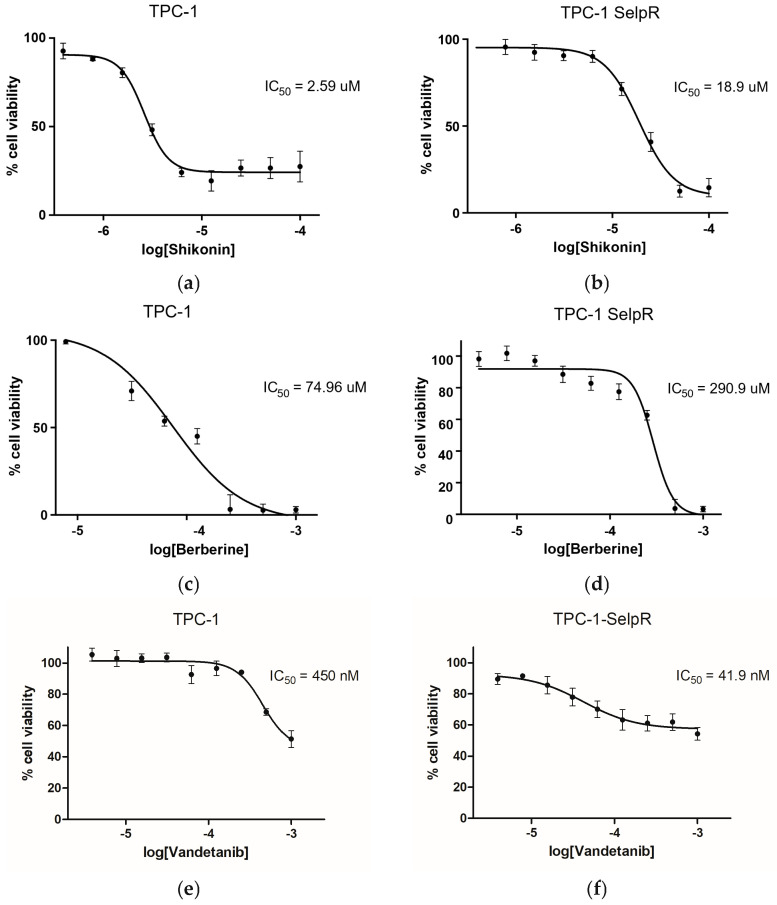
Effects of shikonin, berberine, and vandetanib on cell viability and determination of the IC_50_ in TPC-1 (**a**,**c**,**e**) and TPC-1-SelpR (**b**,**d**,**f**) cell lines.

Since both berberine and shikonin generally affect drug resistance and are closely related to RET and WT1 signaling, resistant cells were treated with several concentrations of both nutraceuticals (ranging from 100 µM to 0.39 µM). The results of the MTT assays showed that the TPC-1 cell line is particularly sensitive to shikonin compared to berberine, with an IC_50_ of approximately 2.59 µM (Figure 13a) compared to 74.96 µM (Figure 13c). In contrast, the TPC-1-SelpR cell line is less sensitive to both nutraceuticals (shikonin, IC_50_ ~18.9 µM; berberine, IC_50_ ~290.9 µM) (Figure 13b,d), although shikonin is more effective, with a lower IC_50_ value, compared to berberine. Interestingly, the IC_50_ value for shikonin increased >7 times in resistant cells, while IC_50_ increased >3 times in the case of berberine. Exploratory analyses then moved on to the previous-generation drug, vandetanib. IC_50_ analyses revealed a value of approximately 450 nM in TPC-1 (Figure 13e) and tenfold lower in TPC-1-SelpR, at approximately 41.9 nM (Figure 13f).

## 4. Discussion

Among the main driver mutations found in PTC are BRAF, RAS, and RET, including CCDC6-RET. For a long time, BRAF, RAS, and RET mutations were considered mutually exclusive, although concomitant cases have been found [46]. A recent case report suggested that CCDC6-RET may serve as a bypass mechanism in EGFR-mutated NSCLC [47]. Given these considerations, we investigated potential mechanisms underlying the RET translocation. To better understand the selpercatinib resistance in PTC harboring CCDC6-RET, a resistant cell line—TPC-1-SelpR—was generated. After prolonged treatment with selpercatinib, the IC_50_ value in the TPC-1 cell line was significantly higher than in control TPC-1 cells, suggesting the onset of drug resistance mechanisms. The analyses of TCGA clinical data, obtained from BioPortal for Cancer Genomics and The Human Protein Atlas, suggest that ex vivo WT1 expression is virtually absent in PTC harboring the CCDC6-RET fusion. This clinical evidence supports the hypothesis that WT1 transcriptional silencing is not a random event, but rather a negative factor in this specific PTC subtype. Resistance to selpercatinib is not associated with on-target mechanisms targeting CCDC6-RET, but rather with off-target mechanisms involving signaling pathways whose inhibition sensitizes. Since selpercatinib interacts with RET and CCDC6-RET, it was assumed that mechanisms closely related to RET were involved in drug resistance. The promoter of the translocation product, CCDC6, was studied; therefore, subsequent analyses focused on potential factors transacting with the RET-fusion promoter. The LASAGNA algorithm identified several transcription factors putatively involved in regulating the CCDC6-RET fusion product, including WT1. Bioinformatic analyses were performed by comparing high-throughput RNA sequencing results from different PTC samples and adjacent healthy tissue. By filtering out the upregulated genes, GO analyses conducted on PTC ex vivo data highlighted that overexpressed genes were involved in cell–cell and cell-extracellular matrix adhesion factors. Furthermore, these analyses described significant enrichments for the WT1 transcription factor, suggesting a close interdependence between WT1 and adhesion factors. Our TPC-1-SelpR model, contrary to expectations, showed a downregulation of WT1. To reproduce the same WT1 gene expression pattern found in TPC-1-SelpR, we partially downregulated WT1 in resistant cells. Since WT1 could be localized to the CCDC6-RET promoter, loss of WT1 function would have led to a modulation in CCDC6-RET driver gene expression, both in TPC-1 and TPC-1-SelpR. Real-time PCR analyses showed distinct expression patterns in both cell lines: siRNA against WT1 in TPC-1 decreased CCDC6-RET gene expression, whereas in TPC-1-SelpR, it increased, suggesting a role for WT1 as a context-dependent modulator. Due to limitations on further direct validation experiments, we cannot establish with certainty whether modulation of CCDC6-RET is directly induced by the WT1 transcription factor, as it may also involve feedback loops or other transcription factors. We observed an increase in CCDC6-RET protein levels in both TPC-1-SelpR and TPC-1-SelpR^WT1-kd^, suggesting that selpercatinib resistance may be subject to strong genetic reprogramming. The discrepancy between mRNA and protein levels is typically observed across several biological contexts, due to distinct regulatory mechanisms between the transcript and its protein product [48,49]. In TPC-1-SelpR^WT1-kd^, Vimentin and Profilin gene expression levels were still high, while Twist and Integrin beta-1 expression significantly increased when compared to TPC-1-SelpR. Confocal microscopy analyses showed that Vimentin distribution was more dispersed in TPC-1-SelpR than in TPC-1. Subsequent cell population analysis demonstrated that the per-cell density of Vimentin expression decreased in TPC-1-SelpR^WT1-kd^. When WT1 is downregulated in resistant cells, the high levels of Vimentin gene expression, as assayed by real-time PCR, do not coincide with the decreased levels observed by Western blot analysis, suggesting the presence of post-transcriptional regulatory mechanisms that uncouple EMT-related transcriptional programs from protein expression, favoring stem-like adaptive states. Of note, downregulation of Vimentin has been shown to confer drug resistance in an ovarian cancer cell model by increasing stemness markers and blocking cells in the G2 phase. Furthermore, its overexpression led to increased sensitivity to cisplatin [50]. It is known that even reduced levels of Vimentin increase cell motility, suggesting that cells are exploiting a deformability-based adaptive state that reduces perinuclear stiffness [51]. Oct4 and Sox2 maintain a stem cell identity, whereas Sox17 promotes a more differentiated phenotype. The partial reduction in WT1 expression in TPC-1-SelpR, induced by siRNA, was accompanied by an increase in the expression of the Oct4 and Sox2 genes, while Sox17 was undetectable. The increased stemness marker Sox2 is also detectable by Western blot analysis, while Sox17 is lost in TPC-1-SelpR^WT1-kd^. Notably, Sox2 is not detectable as a band at approximately 35 kDa but rather as a high-molecular-weight complex (~130 kDa), suggesting the formation of stable, active complexes. Wound-healing results demonstrated that silencing WT1 in TPC-1 WT1 inhibits cell migration, whereas silencing SelpR in TPC-1-SelpR enhances migratory ability. Based on these findings, even considering that our resistant cells grow more slowly, WT1 loss may rewire a plasticity-driven adaptive state. This suggests that the loss of WT1 function manifests as two distinct and opposite behaviors in PTC. In TPC-1, loss of WT1 function assumes an inhibitory role in tumor progression. In TPC-1-SelpR, loss of WT1 function triggers a circuit that promotes CCDC6-RET and consolidates a more invasive stem-like phenotype. Finally, the nutraceuticals berberine and shikonin were considered because they can modulate proteins involved in RET-dependent signaling. Exploratory analyses using berberine and shikonin showed that both nutraceuticals exerted an effect on TPC-1 cell viability, and that shikonin exhibited a significantly lower IC_50_ value than berberine. However, in the TPC-1-SelpR cell line, the IC_50_ levels were significantly increased, appearing less sensitive to the action of both nutraceuticals, suggesting cross-resistance mechanisms. Exploratory analyses, aimed at characterizing potential vulnerabilities of resistant cells, also included vandetanib, an older-generation drug. Unlike the latest-generation drugs, vandetanib is not specific for RET; rather, it inhibits other targets. Vandetanib showed a much lower IC_50_ value in selpercatinib-resistant cells, suggesting collateral sensitivity to the drug, a phenomenon well described [52]. The observed collateral sensitivity to vandetanib suggests that resistant cells may reduce the dependencies from RET-driven oncogenic axis.

## 5. Conclusions

The results obtained suggest that selpercatinib-mediated resistance may be associated with partial, rather than complete, loss of function of the transcription factor WT1. In TPC-1, WT1 knockdown contributes to a tumor suppressor function. The selpercatinib-resistant cell line exhibits reduced WT1 levels and appears more prone to a stem-like shift. Further downregulation of WT1 in the selpercatinib-resistant line consolidates a less differentiated phenotype with stem-like characteristics, promoting an adaptive plastic state that allows them to survive the pharmacological pressure of selpercatinib, thus sustaining resistance. These findings suggest that selpercatinib resistance may involve both on-target and off-target mechanisms. Future experiments could be directed towards comprehensive functional studies to analyze the effects of WT1 gain-of-function in the selpercatinib-resistant line, and towards differential proteome analysis by mass spectrometry to better understand the mechanisms of selpercatinib resistance.

## Data Availability

The original contributions presented in this study are included in the article. Further inquiries can be directed to the corresponding author.
